# Accumulated cognitive impairment, frailty, burden, and perceived stress and the risk of hospitalization and mortality in older caregivers

**DOI:** 10.1590/1980-5764-DN-2020-0091

**Published:** 2022

**Authors:** Allan Gustavo Bregola, Ana Carolina Ottaviani, Bruna Moretti Luchesi, Sofia Cristina Iost Pavarini

**Affiliations:** 1 Universidade Federal de São Carlos, Programa de Pós-Graduação em Enfermagem, São Carlos SP, Brazil.; 2 University of East Anglia, Faculty of Medicine and Health Sciences, Norwich, United Kingdom.; 3 Universidade Federal de São Carlos, Departamento de Gerontologia, São Carlos SP, Brazil.; 4 Universidade Federal de Mato Grosso do Sul, Faculdade de Medicina, Três Lagoas MS, Brazil.

**Keywords:** Survival, Caregivers, Longitudinal Studies, Risk Factors, Sobrevida, Cuidadores, Estudos Longitudinais, Fatores de Risco

## Abstract

Combination of cognitive impairment, frailty, perceived stress, and excessive burden poses a risk to the health of caregivers.

**Objectives:**

This study aimed to investigate the influence of the combination of these conditions on the occurrence of hospitalizations and deaths among older caregivers in a 4-year follow-up period.

**Methods:**

This is a longitudinal study in the communities with 351 older caregivers who underwent gerontological and geriatric evaluations in 2014 and completed cognitive (Mini-Mental State Examination), physical frailty (Cardiovascular Health Study criteria), perceived stress (Perceived Stress Scale), and care burden (Zarit Burden Interview) assessments. In 2018, data on hospitalization and mortality were collected.

**Results:**

As a result, 32 (12.6%) caregivers had deceased. Among the 228 survived caregivers who were reevaluated, 24% reported using hospital services in the previous year. Mean length of hospital stay was 3 days (range: 1–22 days). Hospitalization was associated with cognitive impairment co-occurring with frailty (p=0.05), stress (p=0.03), burden (p=0.01), and frailty co-occurring with stress (p=0.04). Considering singular effects, the mortality rate (33.3%) was higher among frail caregivers, followed by those with cognitive impairment (23.1%) and a high level of perceived stress (20.4%). Considering accumulative conditions, mortality rate (43.8%) was higher among frail older caregivers with cognitive impairment, followed by those with a high level of perceived stress and cognitive impairment (32.4%).

**Conclusions:**

The investigation of accumulated effects is important to the identification of potentially vulnerable older caregivers as well as the management and monitoring of the care, health, and independence of those who provide care for other older adults.

## RESUMO.

Comprometimento cognitivo, fragilidade, estresse e sobrecarga, combinados, podem representar maior risco à saúde dos cuidadores.

**Objetivos:**

O objetivo do estudo foi analisar a influência da combinação dessas condições na ocorrência de hospitalizações e óbitos entre idosos cuidadores em um período de seguimento de quatro anos.

**Métodos:**

Estudo longitudinal na comunidade, com 351 idosos cuidadores submetidos a avaliação gerontológico-geriátrica em 2014, composta de avaliação da cognição (Miniexame do Estado Mental), fragilidade física (critérios do *Cardiovascular Health Study*), estresse percebido (Escala de Estresse Percebido) e sobrecarga do cuidado (*Zarit Burden Interview*). Em 2018, foram coletados dados sobre admissão hospitalar e mortalidade.

**Resultados:**

Trinta e três (12,6%) cuidadores faleceram. Entre os 228 cuidadores sobreviventes, 24% relataram ter utilizado os serviços hospitalares no ano anterior. A hospitalização foi associada com comprometimento cognitivo coocorrendo com fragilidade (p=0,05), estresse (p=0,03) e sobrecarga (p=0,01), bem como fragilidade coocorrendo com estresse (p=0,04). Considerando-se os efeitos singulares, a taxa de mortalidade foi maior entre cuidadores frágeis (33,3%), seguidos daqueles com comprometimento cognitivo (23,1%) e alto nível de percepção de estresse (20,4%). Considerando-se as condições acumuladas, a mortalidade foi maior entre cuidadores idosos frágeis com comprometimento cognitivo (43,8%), seguidos por aqueles com alto nível de estresse percebido e comprometimento cognitivo (32,4%).

**Conclusões:**

A investigação dos efeitos acumulados é importante para a identificação de cuidadores de idosos potencialmente vulneráveis, bem como para o gerenciamento e o monitoramento do cuidado, para a saúde e a independência daqueles que cuidam de outros idosos.

## INTRODUCTION

Due to the greater proneness to chronic diseases associated with both lifestyle and the aging process, the “aging” population experiences heterogeneous changes in levels of functioning. The need for support occurs when an older adult no longer has the resources or cognitive, functional, and behavioral reserves necessary for the maintenance of independence and autonomy. The responsibility normally falls to the closest person in the affective-social circle.

When providing care in old age, caregivers are exposed to syndromes related to their own aging^1^, such as cognitive impairment and physical frailty. In recent decades, such conditions have been determined to be risk factors for a poorer quality of life and a greater likelihood of hospitalization and death^2,3^. The combination of cognitive impairment and frailty, which is denominated as a clinical syndrome involving the simultaneous occurrence of cognitive and physical decline^4^, has been highlighted as the strongest factor associated with the future negative impact on the health of older adults due to the additive effect of the two conditions^5,6^.

In the literature, it is unclear whether levels of perceived stress and caregiver burden are the primary reasons for the need to seek a high-complexity healthcare service, such as a hospital or emergency/urgent care unit. Vascular and coronary disease, cardiopulmonary disease, pneumonia, and stroke are the leading reasons for the hospitalization of older adults^7^. Poor emotional well-being may be a secondary symptom of an adverse health condition and is part of the systematic health illness process^8,9^. This system, with the cited components, is not sufficiently discussed in a complex context of providing care.

The costs of hospitalization for patients aged 60 years or above are considered high, and such individuals are major users of these healthcare institutions^10^. It is important to remember that hospitalization per se is a considerable challenge in the lives of older caregivers and their families. The functioning of the caregiver may not be the same after being discharged from hospital^11^, and an older adult who was previously a provider of care ends up in need of daily assistance. Moreover, hospitalization requires the family to provide resources to fill in for the hospitalized caregiver during the recovery period. While the hospitalization of a caregiver requires coping on the part of the family, the death of a caregiver requires a greater response from the family. One study found that the death of a caregiving spouse occurs less often in comparison with the death of the care recipient, but, when it occurs, it constitutes a major change in the care plans^12^.

A study conducted in Australia with 2,562 older men found that depression was associated with mortality in 4 years and the increase in the mortality rate was associated with frailty^13^. Another study employing meta-analysis on data from 14,302 individuals found that the co-occurrence of cognitive impairment (without dementia) and physical frailty had additive effects on the future diagnosis of dementia^14^. A third systematic review and meta-analysis with >40,000 participants showed that the co-occurrence of risk factors for dementia contributed linearly to the risk of this outcome^15^. Despite the different research methods, different baseline conditions, and different future adverse health conditions in these studies, we suggest that accumulated conditions are strong determinants of health, especially in old age.

However, no longitudinal studies addressing cumulative effects have included older populations or caregivers residing in Brazilian communities or have hypothesized whether aging conditions (e.g., cognitive impairment and frailty) and the care situation (e.g., risk for care burden and perceived stress) could be the determinants of health in this unique population. Thus, this study can contribute to multidisciplinary health care, meeting the need to investigate frailty and cognition in specific populations, such as caregivers, and analyzing the effects on adverse outcomes, such as hospitalization and death.

The aim of this study was to investigate the cumulative effects of the conditions of older caregivers (i.e., cognitive impairment and frailty) and those associated with care (i.e., burden and level of perceived stress) on adverse health outcomes among older caregivers, such as the need for hospitalization in the previous year and the occurrence of death in the follow-up period. Our initial hypothesis is that there are cumulative effects of cognitive impairment, frailty, and depressed psychological well-being.

## METHODS

### Design

This is a longitudinal study with a 4-year follow-up.

### Participants and recruitment process

The participants comprised a population of 351 caregivers aged 60 years or above from a study entitled “Variables associated with cognition in older caregivers” in 2014 (baseline) and a study entitled “Follow-up of older caregivers in primary care” conducted at 18 family health units (FHUs) (i.e., local public primary care modality) in the city of São Carlos. This city is located in the state of São Paulo in the southeastern region of Brazil and has an estimated population of 222,000 residents.

The recruitment process was based on the degree of dependence of the care recipient regarding basic and instrumental activities of daily living, which were, respectively, analyzed using the Katz Index and Lawton and Brody Scale. The inclusion criteria were being a primary caregiver of a dependent older person (≥60 years old) living in the same household. To be considered dependent, the care recipient had to be dependent with regard to at least one activity of daily living. The instruments were also applied to the older caregiver, who needed to be more independent than the care recipient. Baseline data were collected through face-to-face contact. Household interviews were conducted by trained interviewers between April and December 2014.

For the 2018 data collection (follow-up), older caregivers were interviewed at baseline and/or their relatives were invited to participate. Data collection was performed either at the caregivers’ homes or by telephone. Information on mortality was collected from the families and, subsequently, confirmed by the FHU teams. The minimum sample size in the follow-up group was n=154, with an alpha of 0.05 and a power of 0.90, considering the heterogeneity of the population. The follow-up data collection procedures were conducted between 45 and 48 months after the baseline collection.

**Figure 1:**
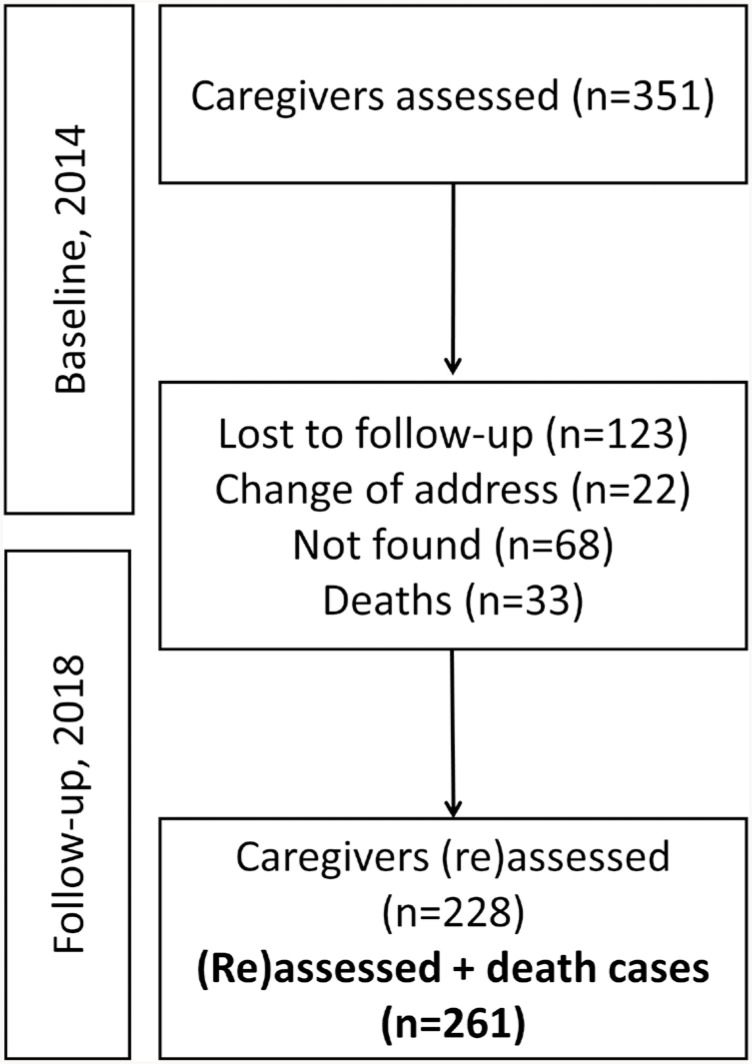
Study participants screening for the longitudinal study. São Carlos, Brazil, 2014–2018.

Among the 351 caregivers evaluated in 2014, 22 had moved from the area of coverage of the FHUs during the follow-up period and 68 were not located after three home visits or by telephone. Thus, the baseline data in this study refer to information from 261 participants, among whom 33 (12.6%) were confirmed death. The remaining 228 individuals were reevaluated for the collection of the follow-up data. Three participants were excluded from the analysis for not completing the cognition, frailty, and stress assessments (Figure 1).

All participants gave their written informed consent in compliance with the Resolution 466/12 from the National Health Council (CNS). This study received authorization from the São Carlos Municipal Secretary of Health (certificate number: 68/2014) and approval from the Human Research Ethics Committee of Universidade Federal de São Carlos (favorable opinion number: 46431315.3.0000.5504).

### Variables and evaluations

#### 2014 evaluations (baseline)

- *Cognitive impairment*: The Mini-Mental State Examination (total score: 0–30 points)^16^ was used for the assessment of cognitive impairment using the following cutoff points adjusted for schooling: <26 points for participants with 9 or more years of schooling; <24 points for participants with 5–8 years of schooling; <22 points for participants with 1–4 years of schooling; and <17 points for participants with no schooling (illiterate)^17^. The revised version of Addenbrooke’s Cognitive Examination (ACE-R)^18,19^ was also used for the sole purpose of comparing scores between groups (Table 1).
- *Physical frailty* was defined using the five components of the frailty phenotype described by Linda Fried of the Cardiovascular Health Study Collaborative Research Group in 2001: (1) unintentional weight loss in the previous year; (2) fatigue or exhaustion during routine activities; (3) muscle weakness (determined by low grip strength measured using a dynamometer); (4) slowness (determined by slow gait speed during a 4.6-m walk); and (5) low level of physical activity, based on the reports of the interviewees. Three or more of the five components of the frailty phenotype were considered indicative of frailty; one or two components were considered indicative of prefrailty, and the absence of criteria indicated that the individual was robust or nonfrail^20^.
- *Caregiver burden* was assessed using the short 12-item version of the Zarit Burden Interview (ZBI)^21,22^, which measures the perceived impact on the physical and emotional health, social aspects, and financial situation of the family caregiver. Each item has five response options, ranging from 12 to 48 points. A cutoff point of ≥13 was used for the identification of caregivers with a higher level of burden^23^.
- *Perceived stress* was measured using the Perceived Stress Scale (PSS), which was developed to measure the level of stress experienced in the previous month. The PSS has 14 items, with 5 response options ranging from “never” to “very often.” The score ranges from 0 to 56 points, with higher scores denoting a higher level of stress. In this study, the median of ≥17 points was used as the cutoff point to define caregivers with high and low levels of stress^24,25^.
- *Demographic characteristics* such as sex (female/male), age (continuous and by range), years of schooling (continuous), family income in Brazilian currency (R$) (continuous), and retirement (yes/no) were evaluated.
- *Care context*: The care-related data such as relationship to dependent care recipient (spouse/others), duration of care in years (continuous and per range of time), daily care hours (range of time), financial/material assistance (receiving financial support, medicine, goods, or supplies to assist in providing care: yes/no), and emotional/affective support (receiving formal psychological help or feeling emotionally supported by others to cope with the challenges of providing care: yes/no) were collected. The age, sex, and dependence level of care recipient were also recorded.

**Table 1:** Baseline, demographic, and care-related characteristics of caregivers. São Carlos, Brazil, 2014.

		Survivors not hospitalized between 2017 and 2018 (n=171)	Survivors hospitalized between 2017 and 2018 (n=54)	Deceased between 2014 and 2018 (n=33)	All caregivers (n=258)
Sex	**Male**	18.1	24.1	42.4	**22.5**
	**Female**	81.9	75.9	57.6	**77.5**
Age (years)		68.9±6.3	69.9±6.7	73.9±9.0	**69.7±6.9**
	**60–69**	59.6	50	39.4	**55.0**
	**70–79**	32.2	38.9	36.4	**34.1**
	**≥80**	8.2	11.1	24.2	**10.9**
Schooling (years)		3.5±3.0	3.4±3.5	4.2±4.4	**3.61±3.33**
	**+9**	8.2	9.3	9.1	**8.5**
	**5–8**	12.3	7.4	9.1	**10.9**
	**1–4**	60.8	57.4	60.6	**60.1**
	**Illiterate**	18.7	25.9	21.2	**20.5**
Monthly family income (R$)		2178±1349	2266±1655	1943.48±936	**2167±1376**
Retired	**Yes**	64.9	68.5	78.8	**67.4**
	**No**	35.1	31.5	21.2	**32.6**
Relationship to care recipient	**Spouse**	84.6	90.7	78.8	**85.3**
	**Others**	15.4	9.3	21.2	**14.7**
Duration of care (years)		10.8±14.0	8.0±10.6	7.4±10.3	**9.8±12.9**
	**<6**	48.5	51.9	48.5	**49.2**
	**≥6**	48.5	46.3	45.5	**47.7**
	**Missing**	2.9	1.9	6.1	**3.1**
Hours dedicated to care per week		38.2±31.4	50.3±41.3	38.8±25.9	**40.9±33.4**
	**<40**	68.4	55.6	54.5	**64.0**
	**>40**	29.2	44.4	39.5	**33.7**
	**Missing**	2.3	–	6.1	**2.3**
Financial support	**Yes**	13.5	7.4	15.2	**12.4**
	**No**	86.0	92.6	84.8	**87.2**
	**Missing**	0.6	–	–	**0.4**
Affective support	**Yes**	46.2	44.4	45.5	**45.7**
	**No**	53.2	55.6	54.5	**53.9**

Data are presented as % or mean±standard deviation.

#### 2018 evaluations (follow-up)

- *Cases of deaths:* For confirmed cases of deaths, information on the cause and date of death were collected from the family and confirmed with the FHU team, offering coverage to the area of the participant’s home.
- *Admissions to hospital among surviving participants:* The participant was asked the following questions: Did you need to be hospitalized or use high-complexity healthcare services, such as an emergency/urgent care unit, for at least 24 h in the last 12 months? If so, how many times and what was the total number of days you were hospitalized?

#### Cumulative conditions (+) and additive effects

- *Cognitive impairment and frailty:* It is defined as the simultaneous occurrence of cognitive impairment and physical frailty (not prefrailty) (reference: cognitively intact and nonfrail caregivers).
- *Cognitive impairment and burden:* It is defined as the simultaneous occurrence of cognitive impairment and a high level of caregiver burden (reference: cognitively intact caregivers without excessive burden).
- *Cognitive impairment and stress*: It is defined as the simultaneous occurrence of cognitive impairment and a high level of perceived stress (reference: cognitively intact caregivers with a low level of perceived stress).
- *Frailty and burden*: It is defined as the simultaneous occurrence of physical frailty (not prefrailty) and a high level of caregiver burden (reference: nonfrail caregivers without excessive burden).
- *Frailty and stress*: It is defined as the simultaneous occurrence of physical frailty (not prefrailty) and a high level of perceived stress (reference: nonfrail caregivers with a low level of perceived stress).

### Data analysis

The data collected in 2014 were entered twice in a databank on Excel 2016 (Microsoft Corp., Redmond, WA, USA) by two independent individuals. The information collected in 2018 was compiled in the baseline database and followed the same data entry procedure. Inconsistencies were checked and corrected. *Statistical Package for the Social Sciences* (SPSS), version 25.0 (IBM Inc., Chicago, IL, USA) was used for the treatment and analysis of the data.

In the presentation of the information (Table 1), the sample was divided into three groups: (1) surviving older caregivers with no report of hospitalization in the previous year, (2) surviving older caregivers with a report of hospitalization in the previous year, and (3) older caregivers who had deceased during the follow-up period. Means, proportions, and dispersion data of the variables collected at baseline (e.g., demographic and care-related characteristics) were presented for each of these subgroups. Pearson’s χ^2^ test, the t-test, and analysis of variance (ANOVA) were performed, and F_df_ was reported.

Binary logistic regression was performed to analyze the factors associated with the hospitalization of the caregivers. Reports of hospitalization were incorporated into the model as the dependent variable. The independent variables were cognitive impairment (reference: absence of cognitive impairment), frailty (reference: absence of frailty), excessive burden (reference: absence of excessive burden), a high level of stress (reference: low level of stress), and the accumulated conditions. *Odds Ratio* (OR), 95% confidence intervals (95%CIs), and p-values are calculated (Table 2).

**Table 2:** Binary regression for hospitalization and Cox regression for mortality of caregivers. São Carlos, Brazil, 2014–2018.

	Hospitalization			Death		
	OR	95%CI	p-value	HR	95%CI	p-value
**Cognitive impairment**, *unadjusted*	1.77	0.91–3.44	0.088	0.80	0.33–1.93	0.624
*Adjusted by sex*	1.82	0.93–3.55	0.076	0.85	0.37–2.03	0.679
*Adjusted by age (median 69 years)*	1.82	0.94–3.56	0.075	0.49	0.71–1.43	0.196
*Adjusted by sex and age (median 69 years)*	1.86	0.95–3.63	0.069	0.51	0.17–1.49	0.218
**Frailty**, *unadjusted*	2.08	0.86–5.03	0.101	1.03	0.24–4.47	0.960
*Adjusted by sex*	1.98	0.81–4.84	0.132	0.54	0.09–3.27	0.503
*Adjusted by age (median 69 years)*	2.50	0.98–6.35	0.054	1.03	0.24–4.47	0.960
*Adjusted by sex and age (median 69 years)*	2.46	0.94–6.39	0.064	0.54	0.08–3.27	0.503
**Stress**, *unadjusted*	1.31	0.70–2.43	0.395	0.81	0.34–1.93	0.639
*Adjusted by sex*	1.31	0.70–2.44	0.392	0.81	0.34–1.93	0.637
*Adjusted by age (median 69 years)*	1.41	0.74–2.68	0.287	0.79	0.33–1.90	0.607
*Adjusted by sex and age (median 69 years)*	1.40	0.73–2.65	0.303	0.79	0.33–1.92	0.617
**Care burden**, *unadjusted*	1.63	0.81–3.26	0.163	**7.06**	**1.18–42.28**	**0.032**
*Adjusted by sex*	1.63	0.81–3.26	0.163	**6.97**	**1.16–41.78**	**0.033**
*Adjusted by age (median 69 years)*	1.59	0.79–3.19	0.190	**6.47**	**1.06–39.42**	**0.043**
*Adjusted by sex and age (median 69 years)*	1.60	0.79–3.21	0.186	**6.30**	**1.03–38.46**	**0.046**
**Cumulative effects of cognitive impairment with:**						
**Frailty**, *unadjusted*	2.89	0.87–9.54	0.082	1.21	0.62–5.66	0.801
*Adjusted by sex*	**3.83**	**1.04–14.07**	**0.043**	1.33	0.22–8.18	0.756
*Adjusted by age (median 69 years)*	3.08	0.88–10.80	0.078	0.81	0.17–3.81	0.801
*Adjusted by sex and age (median 69 years)*	**3.86**	**1.00–14.87**	**0.050**	1.33	0.21–8.18	0.756
**Stress**, *unadjusted*	2.22	0.90–5.45	0.080	0.72	0.24–2.11	0.551
*Adjusted by sex*	2.73	1.06–7.07	0.038	0.74	0.24–2.23	0.597
*Adjusted by age (median 69 years)*	2.24	0.91–5.50	0.078	0.52	0.15–1.84	0.317
*Adjusted by sex and age (median 69 years)*	**2.72**	**1.05–7.04**	**0.039**	0.56	0.15–2.02	0.379
**Care burden**, *unadjusted*	**3.85**	**1.32–11.20**	**0.013**	14.00	0.87–223	0.062
*Adjusted by sex*	**3.92**	**1.34–11.46**	**0.012**	13.02	0.78–217	0.074
*Adjusted by age (median 69 years)*	**3.78**	**1.28–11.10**	**0.015**	9.68	0.59–157	0.111
*Adjusted by sex and age (median 69 years)*	**3.83**	**1.30–11.30**	**0.015**	8.55	0.50–144	0.137
Cumulative effects of frailty with:						
**Stress**, *unadjusted*	2.14	0.80–5.75	0.129	§	§	§
*Adjusted by sex*	**3.42**	**1.08–10.84**	**0.036**	§	§	§
*Adjusted by age (median 69 years)*	2.02	0.74–5.48	0.167	§	§	§
*Adjusted by sex and age (median 69 years)*	**3.27**	**1.01–10.58**	**0.048**	§	§	§
**Care burden**, *unadjusted*	2.48	0.75–8.22	0.135	§	§	§
*Adjusted by sex*	2.79	0.80–9.66	0.104	§	§	§
*Adjusted by age (median 69 years)*	1.72	0.47–6.46	0.400	§	§	§
*Adjusted by sex and age (median 69 years)*	1.93	0.50–7.41	0.336	§	§	§

§denotes analysis not conducted due to the small number of cases. OR: *Odds Ratio*; 95%CI: 95% confidence interval.

Survival analysis (Cox regression) was performed for the analysis of factors associated with the mortality of the caregivers. The event of death was incorporated into the model as the outcome and was controlled for the time of death. The independent variables followed the same inclusion pattern as that in the analysis of factors associated with hospitalization. Hazard ratios (HRs), 95%CIs, and p-values were calculated (Table 2).

**Figure 2:**
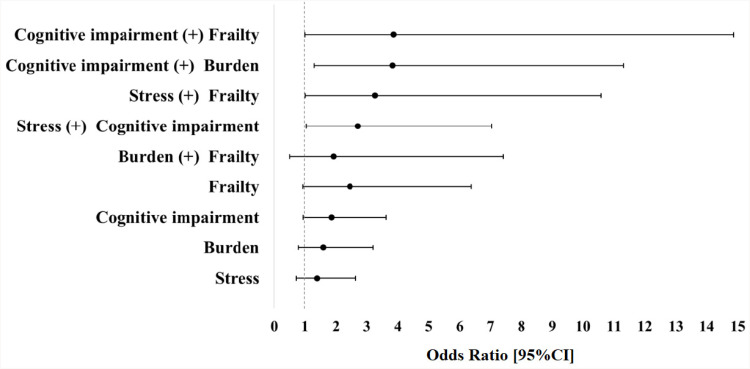
Forest plot for effects of accumulated conditions on hospitalization in older caregivers. X-axis denotes odds ratio with 95% confidence interval (error bars); Y-axis (left side) denotes accumulated conditions tested in association with hospitalization of older caregivers.

**Figure 3:**
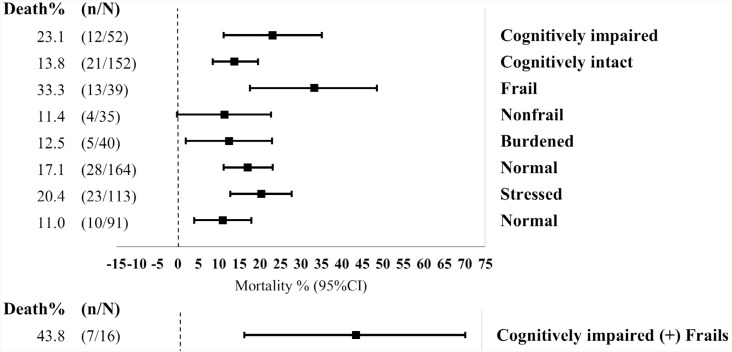
Forest plot of caregiver deaths (%) considering accumulated conditions in older caregivers. X-axis denotes percentages (%) with absolute frequency of caregiver deaths (n) among participants with accumulated conditions; Y-axis denotes plots represent percentages and error bars represent 95% confidence intervals of percentage (%) of cases of caregiver deaths.

Both regression models were adjusted by sex and age. Figure 2 shows the risk factors associated with hospitalization controlled for these two variables. The level of significance was set to be 5% (p<0.05). The independent variables, including accumulated conditions, were transformed into subsamples. The frequencies of mortality and respective CIs were calculated for each subsample and are presented in Figure 3. The calculation of frequency did not include the group of survivors who had been hospitalized.

## RESULTS

### Hospitalization and deaths reports and demographics effects

Among the 351 caregivers in 2014, 90 (25.6%) were lost to follow-up. Of the remaining 261, 33 (12.6%) had deceased and 228 surviving caregivers were reevaluated in 2018. The data from three caregivers were not included in the analyses due to incomplete evaluations at baseline.

Mean age in the overall sample was 70 years in 2014 and 73 years in 2018. The most prevalent age range was 60–69 years in 2014 and 70–79 years in 2018. Among the surviving caregivers in 2018, 54 (20.9%) reported having been hospitalized in the previous year, corresponding to 24% of the surviving caregivers who were not lost to follow-up (n=228).

Table 1 displays the data for the three groups: surviving caregivers in 2018 with no occurrence of hospitalization, surviving caregivers in 2018 who reported hospitalization, and caregivers who had deceased during the follow-up period. The data from 2014 are reported, obeying the division of these groups.

Table 1 shows the predominance of women, the 60- to 69-year-old age group, 1–4 years of schooling, and retired individuals in the three groups. No effect was found for schooling (F_2,255_=0.65; p=0.520) or income (F_2,238_=0.55; p=0.578). The largest percentage of retired participants was in the group of caregivers who had deceased, with a marginally significant difference (p=0.08). A greater effect was found for age (F_2,255_=7.51; p=0.001); caregivers who had deceased were significantly older than the survivors who had not been hospitalized (t=3.85; p<0.001) and were also older than those who had been hospitalized (t=2.33; p=0.02).

### Caregiving effects

Mean age of the care recipients was 73.7±8.4 years, but 22% were 80 years or above. Sixty-nine percent were men, and 12.7% were severely dependent for activities of daily living. Caregivers were predominantly spouses providing care for >6 years and without receiving financial or emotional/motivational support for <40 h/week. A total of 21% of caregivers were not spouses in the group who had deceased, and 9% were not spouses in the group who had been hospitalized; however, this difference did not achieve a statistical significance (χ^2^ test). In the group of surviving caregivers who had not been hospitalized, a mean duration for care was 3 years, with a mean of <0.6 h/week compared with the caregivers who had deceased in 2018 and with a mean of <12 h/week compared with surviving caregivers who had been hospitalized. However, no significant differences between the groups were found for duration of care (F_2,247_=1.51; p=0.223) or weekly care hours (F_2,249_=2.75; p=0.066).

### Cognitive impairment effects

The percentage of participants below the cutoff point for the Mini-Mental State Examination was 36.4, 35.2, and 23.4% in the group of caregivers who deceased, survivors with a report of hospitalization, and survivors with no report of hospitalization, respectively. No significant differences were found when the deceased group (χ^2^=2.45; p=0.091) and hospitalized group (χ^2^=3.90; p=0.054) were compared with nonhospitalized survivors using the χ^2^ test. In comparison with the other groups, hospitalized caregivers had lower cognitive scores on the ACE-R (F_2,255_=2.77; p=0.064) and Mini-Mental State Examination (F_2,255_=2.05; p=0.130), but these differences did not achieve a statistical significance.

### Frailty effects

An increase in the prevalence of frailty was found among the groups: 39.4% of deceased group, 38.9% of hospitalized survivors, and 15.2% of nonhospitalized survivors were frail. The difference in comparison to the nonhospitalized survivors was significant for the deceased group (χ^2^=5.0; p=0.024), but not for the hospitalized group (χ^2^=2.72; p=0.076). Regarding nonfrailty, the frequencies were 12.1, 22.2, and 18.1% for the deceased group, hospitalized survivor group, and nonhospitalized survivor group, respectively.

### Burden and perceived stress effects

Similarly, a high level of stress was more prevalent in the group of caregivers who had deceased (69.7%) compared to hospitalized survivors (59.3%) and nonhospitalized survivors (52.6%), with a marginal difference between the nonhospitalized and deceased group (χ^2^=3.26; p=0.052). The burden of care was more prevalent in hospitalized survivors (29.6%), followed by nonhospitalized survivors (20.5%) and the deceased group (15.2%); however, no significant difference was found among the groups.

### Accumulated conditions (+) effects

The risk of hospitalization was found only when conditions accumulated (+). The accumulation of cognitive impairment with frailty, cognitive impairment with care burden, a high level of perceived stress with frailty, and a high level of perceived stress with cognitive impairment was associated with a greater risk of hospitalization (Table 2; Figure 2). Among the participants who had been hospitalized, the mean length of hospital stay was 3.0±4.0 days (range: 1–22 days). Approximately half (n=29) were hospitalized for 1 day.

The deaths of 33 caregivers occurred between the first semester after the data collection in 2014 and the last semester of the follow-up in 2018. Mean time until death was 2.1±1.0 years after the data collection in 2014. The date of death was recorded in 24 cases, of which 15 (62.5%) occurred in the first 2 years.

In a subsample of comparing those who had not been hospitalized and those who had deceased, the mortality rate (33.3%) was highest among frail caregivers, followed by those with cognitive impairment (23.1%) and those with higher levels of perceived stress (20.4%).

In the analysis of accumulated conditions, mortality rate (43.8%) was highest among frail caregivers with cognitive impairment, followed by caregivers with a high level of perceived stress and cognitive impairment (32.4%), frail caregivers with a high level of perceived stress (32.1%), and caregivers with both cognitive impairment and excessive burden (20%) (Figure 3).

## DISCUSSION

In this study, a transition in age groups occurred between 2014 and 2018, with a greater proportion of individuals aged 70–79 during the second data collection. Among the participants who were reevaluated, about one-quarter reported being hospitalized in the previous year and the percentage of deaths was 12 of 100 caregivers. Caregivers who had deceased during the follow-up period were approximately 4 years older at baseline than those who had survived. The prevalence of the male sex was high among the caregivers who had deceased. The concomitant occurrences of cognitive impairment and frailty, cognitive impairment and a high level of perceived stress, cognitive impairment and burden, frailty and a high level of perceived stress were associated with hospitalization in the previous year. Excessive caregiver burden was associated with mortality. The highest mortality rates were among caregivers with cognitive impairment, frailty, and a high level of perceived stress. Among the participants with accumulated conditions, the prevalence of death was higher among those with cognitive impairment and frailty and those with cognitive impairment and a high level of stress.

This study confirms the findings of previous investigations conducted in Brazil. Caregivers are generally women, who were the spouses of the care recipient, in a similar age range as the care recipient and provide care for many years and many hours per day without receiving any support^26,27^. The lack of support and degree of dependence of the care recipient can considerably increase the level of stress of caregivers, compromising their psychological well-being, cognitive status, and social involvement^28,29^.

Regarding the occurrence of hospitalization, the prevalence in the multicenter *Brazilian Longitudinal Study of Aging* (ELSI-Brazil) was lower than that found in the present investigation (10.2 vs. 24%) in a general population of older adults. A study conducted in the southern region of Brazil with 1,593 older adults found that hospitalization for nonsurgical reasons was more frequent than hospitalization for surgical reasons (17 vs. 10%)^30^. None of the studies cited offered data on the frequency of hospitalizations among older caregivers; however, particularities are known to exist in the different regions of the country.

Women were the majority in the group of caregivers who had been hospitalized, which differs from data reported in a previous study conducted with older adults who lived in nursing homes^31^. In the present investigation, hospitalized caregivers had a higher family income, took care of spouses more, and had been providing care for a shorter period of time. However, they provided care for more hours per week and had no financial or emotional support. These characteristics compose the care context and exert an influence on the occurrence of higher levels of stress and caregiver burden.

The results show that a high level of perceived stress combined with cognitive impairment was associated with the risk of hospitalization. Stress alone is considered a predominant symptom in hospitalized individuals. Indeed, a previous study found that three-quarters of the hospitalized participants had symptoms of stress and more than 10% were in the pre-exhaustion and exhaustion phases caused by psychological symptoms^32^.

We found no studies analyzing the characteristics of being a caregiver and the possible association with admission to hospital, which limits the interpretation on this topic. However, cognitive impairment and frailty are clearly described as factors associated with the risk of hospitalization. From the analysis, cognitive impairment was associated with hospitalization in a multicenter study conducted in France^33^. A meta-analysis with eight studies, many of which used the criteria of the Cardiovascular Health Study, found a clear association between prefrailty/frailty and the risk of hospitalization in older adults^34^.

We also found no studies on the accumulation of conditions. However, some investigations have performed a similar analysis with other variables that are potentially related to this issue. The association between functioning and annual admission to hospitals was only found among older adults with multimorbidity in one study^35^. Functional limitation and morbidity are closely associated with cognitive impairment and physical frailty, as described in previous studies^30,36^.

With regard to mortality, 9% of caregivers had deceased (16.7% when calculated without the group of survivors who had been hospitalized). Among the deaths, 42.4% were men, whereas only 18.1% of the survivors were men. In the group of caregivers who had deceased, there were a larger number of participants aged 80 years or older. The highest mortality rates were found for caregivers with cognitive impairment, frailty, and a high level of perceived stress as well as the accumulation of these conditions.

The mortality rate was similar to that reported in a previous 4-year longitudinal study with caregivers (12%)^37^. In the investigation cited, caregiver strain increased the risk of mortality by 63%. This is in agreement with the present findings, in which the mortality rate was lower among caregivers with low levels of stress and burden.

Feelings of stress in life are reported to be a predictor of mortality in caregivers. In the literature, the risk of mortality in caregivers is lower compared to noncaregivers but increases in the occurrence of reports of psychological stress. In a previous study involving 1,143 older men, feelings of stress experienced throughout the course of one’s life increased the risk of death, with an OR of 1.42 for moderate stress and 1.37 for high stress; moreover, participants with feelings of stress had a 50% greater chance of dying in the follow-up period^38^. In another follow-up study with 375 caregivers of older relatives or friends compared to 694 noncaregivers, the adjusted ratio for the risk of dying in the first 3 years was 0.74 but increased to 1.81 among caregivers with high levels of stress, equaling the risk of mortality found among the noncaregivers^39^.

Excessive caregiver burden alone was associated with the risk of mortality in the Cox regression, but this effect was not found for the other conditions. This finding is likely due to the time component of the event used for the calculation of the hazard ratio, which was better suited for this variable, but the interpretation is limited due to the small number of participants. The broad range of the CI suggests a small number of events (deaths) analyzed in the participants.

Cognitive impairment and frailty increased the percentage of deaths among the caregivers. In the literature, cognitive impairment and frailty have been confirmed as independent factors for mortality in old age^40^. In a study conducted with an initial sample of 1,815 older residents of Latin American heritage in the United States, 690 (38%) deaths occurred after 10 years, and the frequencies of cognitive decline and frailty were relatively higher in these cases compared to the frequencies found among the survivors^40^. In an epidemiological survey involving 2,375 Singaporean Chinese individuals aged 55 years or older and without dementia or neurodegenerative diseases, the participants with concomitant prefrailty and cognitive impairment had a 1.8-fold greater risk of mortality in the 3-year follow-up period; in cases of the concomitant occurrence of cognitive decline and frailty, the risk of mortality increased fivefold^41^. This effect was also found in another study, in which the OR of death in older adults with concomitant cognitive decline and frailty was 1.55-fold higher compared to the analyses in which only frailty was used as the predictor^5^.

A limitation and potential bias of this study was the impossibility of performing a clinical diagnosis of dementia and mild cognitive impairment. However, the neuropsychological test employed (Mini-Mental State Examination) for cognitive screening was used with cutoff points based on the respondent’s level of schooling. For the determination of the co-occurrence of cognitive impairment and frailty, future studies should combine different cognitive tests, different forms of assessing frailty, and the acquisition of subjective data.

The investigation of the effects on health of cognitive impairment, frailty, and the psychological aspects of providing care for a dependent older adult is particularly relevant to the identification of risk factors and the planning of interventions directed at caregivers, especially in the primary care. Such care is commonly provided for a spouse or loved one in their own home for many years and even decades^28,42^. Over time, the functional dependence of the care recipient can increase, with a consequent reduction in autonomy, leading to greater feelings of burden and poorer psychological well-being for the caregiver^28^. Thus, in addition to the conditions of their own aging (decline in cognitive function and physical frailty), caregivers have a greater chance of becoming vulnerable. Indeed, caregivers with accumulated conditions are at greater risk of adverse outcomes compared to healthier caregivers with less psychological burden.

The occurrence of hospitalization was high in this study, and the frequency of deaths among the caregivers was similar to rates described in the literature. Hospitalization and death during the follow-up period were more frequent among the caregivers with cognitive impairment, frailty, a high level of stress, and excessive burden in this specific population. Despite the significant findings reported in this study, we are not able to extrapolate the results to the Brazilian population due to its demographic, economic, social, and cultural diversity. Therefore, it is important to investigate these issues in other regions of the country as well as in other low- and middle-income countries and increase the sample size wherever possible respecting the scientific method. The present findings can contribute to health promotion programs for caregivers to ensure that they remain active and independent in their activities and support that accumulating conditions are a sign of health and safety risk for those more vulnerable and a response is required.
